# Cholesterol-dependent plasma membrane order (L_o_) is critical for antigen-specific clonal expansion of CD4^+^ T cells

**DOI:** 10.1038/s41598-021-93403-5

**Published:** 2021-07-07

**Authors:** Soumini Sengupta, Ritesh Karsalia, Amanda Morrissey, Anil K. Bamezai

**Affiliations:** 1 Department of Biology, Villanova University, 800E Lancaster Avenue, Villanova, PA 19085 USA; 2 Present Address: Rockland Immunochemicals, Limerick, PA 19468 USA; 3 Present Address: MD Program, University of Pennsylvania School of Medicine, Philadelphia, PA USA; 4 Present Address: MS Program, Villanova University, Villanova, PA USA

**Keywords:** Immunology, Lymphocytes, Signal transduction

## Abstract

Early “T cell activation” events are initiated within the lipid microenvironment of the plasma membrane. Role of lipid membrane order (L_o_) in spatiotemporal signaling through the antigen receptor in T cells is posited but remains unclear. We have examined the role of membrane order (L_o_)/disorder (L_d_) in antigen specific CD4^+^ T cell activation and clonal expansion by first creating membrane disorder, and then reconstituting membrane order by inserting cholesterol into the disordered plasma membrane. Significant revival of antigen specific CD4^+^ T cell proliferative response was observed after reconstituting the disrupted membrane order with cholesterol. These reconstitution experiments illustrate Koch’s postulate by demonstrating that cholesterol-dependent membrane order (L_o_) is critical for responses generated by CD4^+^ T cells and point to the importance of membrane order and lipid microenvironment in signaling through T cell membrane antigen receptors.

## Introduction

Sensing a foreign antigen by a clonotypic CD4^+^ T lymphocyte through its antigen receptor, and signal transduction through multiple antigen receptor-associated signaling molecules occurs in lipid microenvironment of the plasma membrane^1–3^. Additionally, co-stimulatory protein, CD28, and other accessary/adhesion proteins initiate signaling pathways/networks driven by many protein tyrosine kinases (such as p56^lck^), adaptor proteins (LAT), small GTP binding proteins^1–3^. These signaling events initiated at the plasma membrane activate three transcription factors, NFκB, NFAT and AP-I, that are critical for promoting survival, clonal expansion, and differentiation of CD4^+^ T cells^2–4^. While the temporal sequence of phosphorylation events, and multiple signaling pathways/networks in T cells has remained the focus of investigations for decades, the role of lipid microenvironment and cholesterol-dependent membrane order in spatial organization of signaling proteins in the plasma membrane in CD4^+^ T cell, and its clonal expansion after sensing foreign antigen remain unclear.

The composition of plasma membrane endows physical properties to it. Experimentation with synthetic/model membranes, either constructed with saturated/unsaturated lipids and cholesterol (GUV) or derived from the cell membranes (GPMV) have provided insights into the associative behavior of its constituent phospholipids^5–11^. The unsaturated and saturated phospholipids visually segregate into the disordered (L_d_) and ordered (L_o_) phases, respectively^5–11^. However, functional significance of such domains in asymmetric biological cellular membranes maintained at 37 °C remains contentious^12–15^. Recent reports show the antigen receptors are uniformly distributed on the plasma membrane of naïve T cells and the monomeric TCRαβ engage the MHC–peptide complexes^16^, suggesting their exclusion from the nanometer scale lipid domains. Consistent with this observation is the data that early T cell signaling events occur outside the lipid nanometer sized domains^17^. In contrast, it is posited that the cellular membranes show assemblies of small dynamic nanometer size ordered domains^18–23^. Small dynamic lipid rafts are stabilized when the proteins/molecules housed in ordered domains are cross-linked to generate meso-scale lipid rafts^18–23^. Additionally, direct interaction between CD4^+^ T cells with the antigen presenting cells promote coalescing of ordered domains^24^. Proposed models of T cell activation need to take into consideration the role of membrane lipid microenvironment, especially the role of cholesterol in contributing to the formation of L_o_ and L_d_ domains in the membrane. Associative behavior of saturated phospholipids is likely to promote ordered domains in live eukaryotic cell plasma membrane, like what is reported in model/induce membranes^5–11^.

We have examined the role of cholesterol-dependent membrane order in antigen-specific responses by primary CD4^+^ T cells. Antigen-specific response in CD4^+^ T cells was assessed after first reducing their membrane order with 7-ketocholesterol and then reconstituting the cholesterol-dependent membrane order by inserting graded amounts of cholesterol. Di-4-ANEPPDHQ, a polarity sensitive fluorescent dye, was used for spectral imaging of the membrane and to quantify, on per cell basis, the membrane order by flow cytometer. The lipophilic fluorescent probe inserts into the outer membrane and reports lipid packing after sensing hydration in membrane. We show the revival of antigen specific CD4^+^ T cell clonal expansion after reconstituting cholesterol-dependent membrane order.

## Methods

### Mice

Four to eight weeks old DO11 TCRαβ transgenic^25^ were used for the experiments reported here. Mice were housed and bred within the Villanova vivarium according to institutional animal care and use committee (IACUC) approved protocol. Ethical approvals for use of mice in experimental studies presented in this manuscript was approved by the IACUC at Villanova University.

### Cell preparation and cell culture

Single cell suspension of lymph node (peripheral, axillary, and mandibular) cells from DO11 mice was made after grinding the tissue using frosted end of glass slides. Extracted cells were filtered through a 100 µm sieve (BD Biosciences, San Jose, CA, USA) and resuspended in RPMI 1640-based wash media supplemented with 5% FBS (Sigma Aldrich, MO, USA) and 2 mM HEPES buffer (Sigma Aldrich, MO, USA). Cells were counted, centrifuged at 1000 RPM for 10 min at 4 °C. Cell pellets were re-suspended at a final concentration of 1 × 10^6^ cells/ml in RPMI 1640-based culture media supplemented with 10% FBS, 2 mM of non-essential amino acids, 50 IU/ml penicillin and 50 μg/ml streptomycin and 1 μg/ml fungizone, 2 mM HEPES (Invitrogen-Life Sciences, Grand Island, NY).

### Insertion of 7-ketocholesterol and cholesterol in the plasma membrane

To generate membrane disorder lymph node cells isolated from DO11 TCR transgenic mice were centrifuged and resuspended in Opti-MEM Media (Invitrogen-Life Sciences, Grand Island, NY), at 1 × 10^6^/ml cell concentration before incubating with 7-ketocholesterol (7-KC) Methyl-beta-cyclodextrin (MβCD) (Sigma Aldrich, MO, USA) complexes for 10 min at room temperature as previously described^26^. The 7-KC and MβCD complexes were generated by mixing each of the ethanol soluble 7-KC at 70 µM, 35 µM and 17.5 µM solutions with water soluble 0.3 mM MβCD. Cholesterol-MβCD complexes were generated similarly. Insertion of oxysterols (and cholesterol) into the plasma membrane is facilitated by MβCD^27,28^. Incubation with 7-KC- MβCD (and cholesterol-MβCD) complexes, was carried out in Opti-MEM to reduce the exposure to serum cholesterol. To restore membrane order, cholesterol (35 µM & 17.5 μM) MβCD (0.3 mM) (Sigma Aldrich, MO, USA) complexes were added to cells following (soon after) the addition of 7-KC-MβCD complexes and incubated for 10 min at room temperature.

### Assessment of membrane order and disorder by flow cytometer

Lymph node cells were incubated with 0.5 µM (final concentration) of Di-4-ANEPPDHQ and anti-CD90.2-Alexa Fluor-647, for 20 min at room temperature (Invitrogen—Life Technologies, Grand Island, NY, USA) as reported before^26^. T cell were enumerated using anti-CD90.2-Alexa Fluor-647 conjugate (Bio Legend, CA, USA). Polarization-resolved measurements in the plasma membrane of the enumerated T cells was assessed by flow cytometer (BD Biosciences, East Rutherford, NJ, USA) as reported before^26^. Briefly, after labeling with Di-4-ANEPPDHQ and anti-CD90.2-AlexaFluor-647 at room temperature (~ 69°F), the probes were washed with isotonic 0.1 M PBS. The membrane dye Di-4-ANEPPDHQ which aligns parallel to the phospholipids in the membrane to report, when excited by 488 nm laser allows polarization-resolved imaging of the plasma membrane, the higher membrane fluidity or disorder (L_d_) at 630 nm and condensed or membrane order (L_o_) at 570 nm are reported^29–33^. Emission from Di-4-ANEPPDHQ fluorophore was captured in FL2 (570 nm) and FL3 (630 nm) channels at room temperature (~ 69°F) after appropriate compensation.

Emission in the range of 670 wavelength from Alexa Fluor-647 on CD90.2 positive cells was captured in FL4 channel of FACSCalibur after appropriate compensation. Untreated lymph node cells the “No treatment group” served as a negative control and cells treated with 0.3 mM MβCD served as a vehicle loading control for all experiments. To quantify alterations in membrane order/fluidity we have used a modified version of the previously published equation to calculate relative (r) GP values to assess overall membrane order/disorder in Di-4-ANEPPDHQ labeled immune cells^31^. Briefly, the GP values were calculated by expressing a normalized ratio of the two Di-4-ANEPPDHQ fluorescence emissions at 570 and 630 nm, reporting overall (bulk) membrane order and disorder, respectively. Flow cytometric gating was set up as previously published^26^. GP was calculated for each sample (10,000 cells), with majority in the gated cohort. While previous reports use two photon microscopy data to assess lipid packing in the plasma membrane after labeling it with Di-4-ANEPPDHQ^31^, our quantification using flow cytometric analyses is on single cell basis.

$$rGP = {\text{~}}\frac{{MFI_{{570}} - {\text{~}}MFI_{{630}} }}{{MFI_{{570}} + {\text{~}}MFI_{{630}} }}$$

rGP = ratiometric Generalized Polarization, MFI = Mean Fluorescence Intensity.

### Live cell imaging of Di-4-ANEPPDHQ stained cells by confocal microscope

To rule out the internalization of the dye confocal imaging was performed following published protocol^34^. DO11 TCRαβ Transgenic mouse lymph node T cells (1 × 10^6^/ml concentration) in Opti-MEM serum-free media were allowed to bind poly-l-lysine coated glass slides (Electron Microscopy Sciences, PA, USA) for 60 min at 4 °C. Excess cells were washed by dipping the slide in Hanks Balanced Salt Solution (HBSS) (Thermo Fisher, MA, USA) at room temperature, also allowing slides to reacclimatize to the ambient temperature. Cells adhered to the glass slide were treated with either 35 μM 7KC (50 μl) or left untreated for 10 min at RT. Excess of 7KC was removed by dipping the slide in HBSS prior to incubation with 5 μM Di-4ANEPPDHQ for 20 min at room temperature or 4 °C. In some experiments, cells attached to poly-l-lysine slides were first fixed with 4% paraformaldehyde (PCHO) phosphate buffer solution (Electron Microscopy Sciences, PA, USA) for 5 min at room temperature before staining with Di-4ANEPPDHQ. Excess of Di-4ANEPPDHQ dye was removed by tipping the slide prior to the mounting step. Stained cells were covered with10 μl of mounting media (Vector Labs, CA, USA) containing DAPI and then covered by glass slide. Cells were imaged using a Leica TCS SP8 inverted confocal fluorescence microscope equipped with standard and HyD PMT's at 630 × total magnification and set to a sequential scan. Previously described confocal equipment setup, with minor changes described below, was used for image acquisition^34^. Samples were illuminated with 408 nm (DAPI) and 488 nm (Di-4-ANEPPDHQ) lasers. DAPI was detected with a standard PMT with a wavelength range of 410–460 nm. Di-4-ANEPPDHQ was collected with two HyD PMTs, the first detecting wavelengths in the range of 500–540 nm (Ordered Phase). The second HyD PMT collected wavelengths in the 640–750 nm range (Disordered Phase). The image size was 512 × 512 pixels and pinhole adjusted to 1 Airy Unit. Scan speed was 400 Hz and line average set at 4. Gains for HyD2 (Ordered) and HyD3 (Disordered) were set to a moderate level. Zoom was set to 1. The Ordered phase was pseudo-colored green while the Disordered phase was pseudo-colored red.

### Assessment of T cell proliferation by MTT assay

Antigen specific clonal expansion/responses of CD4^+^ T cells was assessed using MTT assay kit (Promega Corporation, Madison, WI, USA)^35^. Antigen-specific clonal expansion of untreated or 0.3 mM MβCD treated CD4^+^ cells, 7-KC and/or cholesterol treated CD4^+^ T cells were examined for their potential to clonally expand in the presence of a stimulatory peptide c-Ova_323–339_ (test) and c-Ova_324–334_ (control) peptide (Sigma-Genosys, Woodlands, TX) presented by syngeneic antigen presenting cells. MTT (3-(4, 5-dimethylthiazol-2-yl)-2, 5-diphenyltetrazolium bromide) reagent (20 µl) was added to the cell cultures in 96 titer plate followed by incubation for 4 h in a 37 °C incubator and optical density was measured at 490 nm by spectrophotometer^26^.

### Statistical analysis

ANOVA analysis with Tukey–Kramer post-test analysis were used to determine significance in all experiments. ANOVA analysis were used to find out the differences among group means, while the Tukey–Kramer post-test analysis quantified these differences. Null hypothesis was rejected if p value was < 0.05.

### Ethical approvals and consent to participate

Use of mice in experimental studies presented in this manuscript was approved by the institutional animal care and use committee (IACUC) at Villanova University. All methods used in this study were carried out in accordance with mandatory institutional, State, and Federal regulations and guidelines. Additionally, the study was carried out in compliance with guidelines consistent with the ARRIVE guidelines (http://www.nc3rs.org.uk/page.asp?id=1357).

## Results

### Cholesterol restores plasma membrane order in cells with 7-KC-induced disorder

7-KC, an oxysterol, when delivered to the membrane of CD4^+^ T cells alters membrane order and its ability to proliferate in response to a foreign antigen without compromising the viability of T cells^26^. Revival of antigen-specific responses in T cells after reconstituting membrane order by inserting cholesterol remain untested. To investigate and quantify cholesterol-dependent shifts in the equilibrium from disordered to a more ordered phase, we first developed an experimental system for controlled disruption and reconstitution of membrane order in the plasma membrane of CD4^+^ T lymphocytes. Lymph node cells were first treated with 7-KC (70 µM, 35 µM, 17.5 µM) and then reconstituted with cholesterol at different concentrations (35 µM, 17.5 µM) or left non-reconstituted. Cholesterol, and its derivatives, due to their amphipathic nature, cannot be directly inserted into the plasma membrane and therefore were complexed with water soluble MβCD for membrane delivery^27,28^. MβCD at low concentrations does not alter activation (Supplementary Fig. 1A) and antigen-specific proliferation of CD4^+^ T cells (Supplementary Fig. 1B). To assess membrane disorder in T cells, lymph node cells were stained with Alexa Fluor 647 anti-mouse CD90.2 and fluorescent Di-4-ANEPPDHQ dye followed by incubation with MβCD (0.3 mM) alone or 7-KC (70 µM–17.5 µM)—MβCD complex at room temperature for 10 min. To ensure that Di-4-ANEPPDHQ fluorescent dye binds to the membrane and reports membrane order/disorder in our flow cytometry experiments, which is typically carried out at ambient temperature, we examined the stained cells by confocal microscope. As control we stained cells with Di-4-ANEPPDHQ at 4 °C, a temperature that prevents endocytosis. Supplementary Fig. 2 shows that majority of the dye is bound to the plasma membrane. Moreover, we did not observe evidence of bulk internalization in the absence (Fig. 2S, panels B & C vs N & O) or presence of 7-KC (Fig. 2S, panels F & G vs R & S).

**Figure 1:**
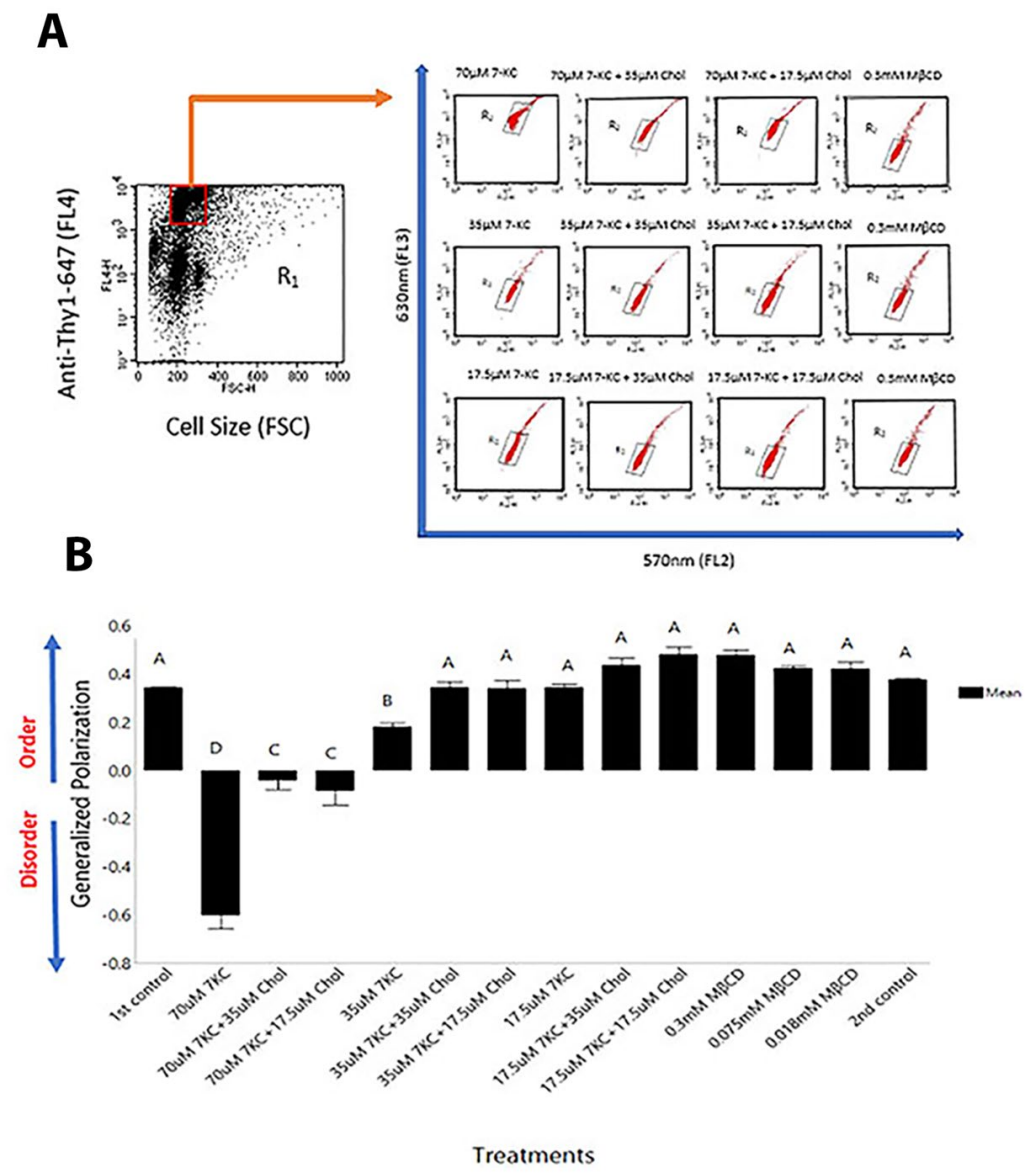
Assessing membrane order and disorder in T cells after staining with di-4 ANEPPDHQ fluorescent dye. Lymph node cells were exposed to di-4 ANEPPDHQ dye and stained with anti-Thy-1 Alexa Fluor 647. Thy-1-positive T cells were enumerated in 7-KC and/or different concentrations of Cholesterol and MβCD-vehicle control were gated (red box) to enumerate T cell subset. Emission at 570 nm and spectral shift to 630 nm, shown as mean fluorescence indices (MFI) registered in FL2 and FL3 channels, report membrane order and disorder, respectively. Representative experiment dot plots showing ordered (X-axis) and disordered (Y-axis) membrane bearing T cells (**A**—upper panel) and quantification of order and disorder as represented by rGP values for different treatment groups (**B**—lower panel) is shown. Control samples (not treatment group) were analyzed at the beginning (1st control) and at the end (2nd control) of the experimental run. All data were computed from 5 independent trials. Error bars represent Standard Error. Statistical significance between the groups was computed by two-way ANOVA and post hoc Tukey analysis using JMP program. Groups with dissimilar connecting letters are significantly different from each other. (p < 0.0001).

For flow cytometery experiments, disruption of membrane order in the enumerated T cell population was quantified after exciting the membrane-bound Di-4-ANEPPDHQ dye at 488 nm and measuring its emission at 570 (FL2 channel) and 630 (FL3 channel) nm by flow cytometer. T cells stained with anti-Thy-1 antibody conjugated with Alexa Flour 647 was excited with 613 nm red laser and emission from this fluorophore was at 668 nm (FL4 channel). Changes in membrane order were quantified on per cell basis using the ratio-metric GP formula, where a negative value indicates “disorder” and positive value reflect “order” in the membrane. Untreated lymph node cells labelled with Di-4-ANEPPDHQ dye and anti-Thy-1 Alexa 647 served as control with 0.35 ratio-metric Generalized Polarization (GP) value. The mean GP value of 70 µM 7-KC treated cells was -0.6, whereas reconstitution of 70 µM treated cells with 35 µM and 17.5 μM Cholesterol showed significantly lower disorder (-0.05 and -0.1 average GP value), respectively (Fig. 1A,B). Cells treated with 35 μM 7-KC exhibited average GP value of 0.2, which when reconstituted with 35 µM and l7.5 μM Cholesterol scaled-up to about 0.35 on GP scale (Fig. 1A,B). Minimal membrane disorder was generated with l7.5 μM 7-KC (0.35 GP value), which when reconstituted with 35 µM and l7.5 μM Cholesterol showed a change to 0.4 and 0.45 on GP scale, respectively (Fig. 1A,B). In contrast, 0.3 to 0.018 mM MβCD vehicle control treated cells was comparable to the control (untreated cells) (Fig. 1B; 0.35–0.4 GP value). These data indicate that 70 µM 7-KC dramatically shifted the equilibrium from an ordered phase to disordered phase, addition of 35 µM cholesterol reconstituted the membrane order significantly, but the reconstituted membrane order was considerably lower than the membrane order of “untreated” and the “vehicle” (0.3 mM MβCD treated cells) control groups (Fig. 1A). Taken together our data shows that T cell membrane order is altered after exposure to 7-KC in a dose dependent manner and significant reconstitution of membrane order was only observed in cells treated with 35 µM cholesterol, whereas reconstitution with l7.5 μM cholesterol induced minimal effect.

### Restoration of antigen-specific T cell responses after reconstitution of membrane order

**Figure 2:**
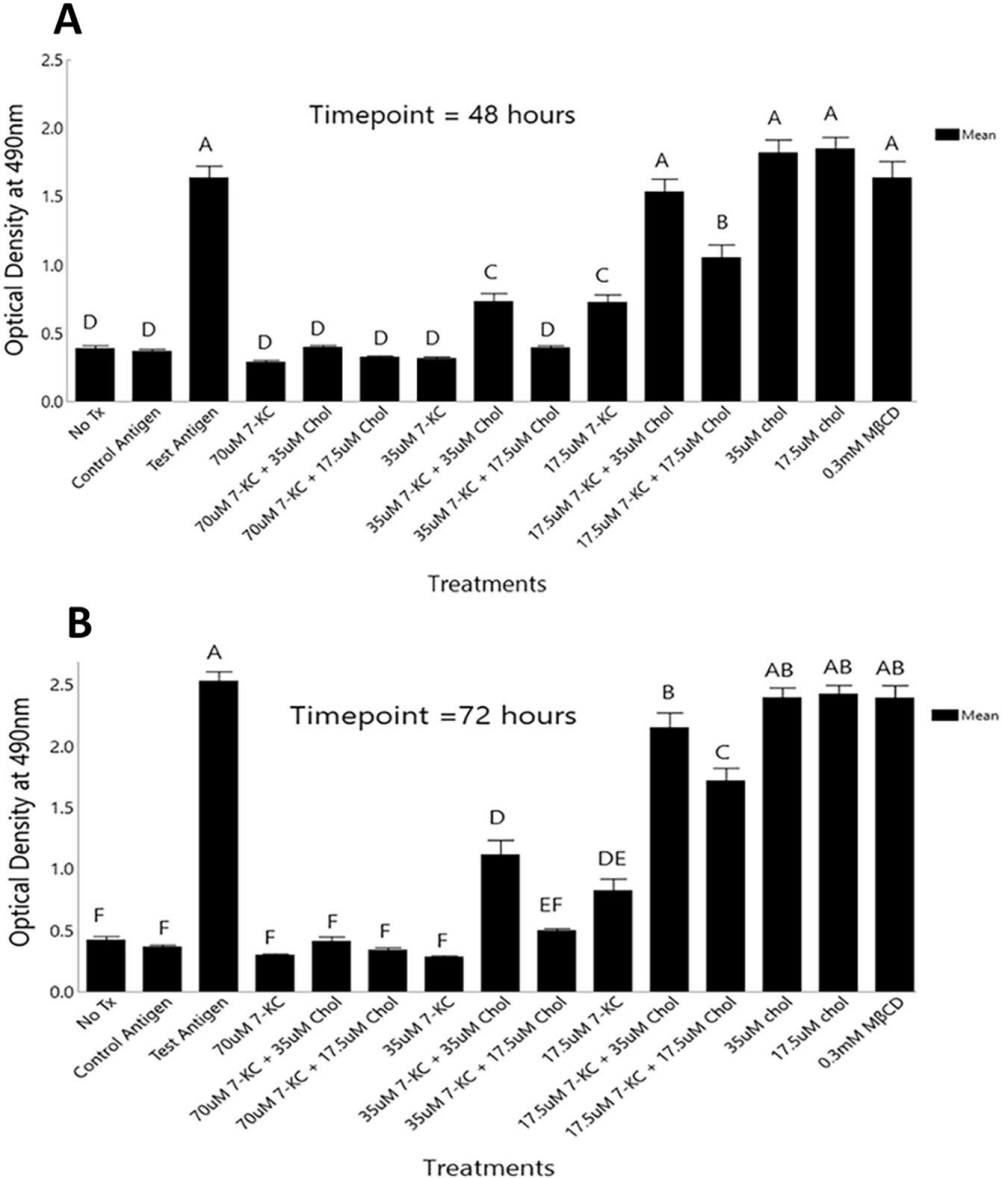
Antigen-specific proliferative responses by CD4^+^T cells with ordered and disordered membranes. Lymph node cells were treated with different concentrations of 7-KC-MβCD complex and/or different concentrations of Cholesterol-MβCD complex and MβCD-vehicle control for 10 min at RT. Proliferative response to stimulatory peptide c-Ova _323–339_ or a control peptide c-Ova _324–334_ were examined after 48 (**A**) and 72 (**B**) hours of incubation at 37 °C incubator. 20 µl of MTT reagent was added to the cell cultures and incubated over the last 4 h of the total incubation time. Optical density of each culture well, that was reflective of the antigen specific CD4^+^ T cell proliferative response, was read at 490 nm using a 96 well plate reader. All data were computed from 5 independent trials. Error bars represent Standard Error. Statistical significance between the groups was computed by two-way ANOVA and post hoc Tukey analysis using JMP program. Groups with dissimilar connecting letters are significantly different (p < 0.05) from each other.

We next examined the role of lipid raft-based membrane order in antigen-specific clonal expansion of CD4^+^ T cells. 7-KC and/or Cholesterol treated lymph node cells were incubated with either stimulatory c-Ova_323–339_, or control c-Ova_324–334_, peptide for 48 and 72 h. We used colorimetric MTT assay to quantify proliferative responses in CD4^+^ T cells. MTT assay measures metabolic activity that tightly co-relates to the numbers of live and proliferating cells in the cell culture^36,37^. Figure 2 shows, in response to c-Ova_323–339_, the lymph node cells treated with 70 µM 7-KC proliferated about fourfold less than the untreated control cultures when assessed at 48-h time point. Reduced proliferation observed with 70 µM 7-KC treated cells resembled the response in cell cultures without antigen or in the presence of non-stimulatory control peptide c-Ova_324–334_ (Fig. 2A). Lower concentrations of 7-KC inhibited cOVA_323–339_ peptide specific proliferative responses in DO11 CD4^+^ T cells in a concentration-dependent manner (Fig. 2A), with the 17.5 µM 7-KC exposure showing only ~ 0.5-fold lower antigen-specific proliferative response than the control group which did not receive 7-KC treatment (Fig. 2A). Lymph node cells previously exposed to 70 µM 7-KC followed by reconstitution of their membrane order with 35 µM cholesterol did not significantly revive proliferative response when compared to non-reconstituted 70 µM 7-KC treated cultures (< 0.1-fold higher proliferation; p value > 0.5) (Fig. 2A). In contrast, 35 µM Cholesterol significantly (p value < 0.001) reconstituted proliferation in cells previously exposed to 35 µM and 17.5 µM 7-KC, increasing proliferation by 1.7 and 1.1-fold (respectively) than the responses generated in the absence of cholesterol (Fig. 2A). Minimal change in proliferation was observed upon reconstitution with 17.5 µM cholesterol. This was the lowest concentration used for reconstituting membrane disorder after treating cells with 7-KC (70 µM, 35 µM, 17.5 µM (Fig. 2A). T cell proliferative response to c-Ova_323-339_ peptide in 0.3 mM MβCD vehicle control treatment group resembled the untreated control group (in the absence of 0.3 mM MβCD), these control experiments demonstrate the specific effects of 7-KC ± cholesterol. Loading extra cholesterol in cells (35 µM & 17.5 µM cholesterol treatment groups), in the absence of any previous exposure to 7-KC, did not show enhanced proliferative responses (Fig. 2A). These responses showed similarity to the response generated in the presence of stimulatory c-Ova_323–339_ peptide alone but were significantly higher than the “no treatment group”, and cultures that received c-Ova_324–334_ control peptide. The latter two groups served as negative, and specificity controls. CD4^+^ T cells with cholesterol-reconstituted membrane order, in response to c-Ova_323–339_ peptide, showed reconstituted proliferation at 72-h time point. The response trend and dosage effect followed the pattern observed at 48 h’ time point. Briefly, in response to c-Ova_323–339_, 70 µM 7-KC treated lymph node cells showed comparable proliferation to the two control cultures, one with c-Ova_324–334_ non-stimulatory peptide, and second in the absence of an antigen (Fig. 2B). Additionally, 7-KC treated cells, in response to the stimulatory c-Ova_323–339_ peptide, proliferated 7.3-fold lower than the cells left untreated. The c-Ova_323–339_ peptide-specific responses generated by DO11 T cells treated with 35 µM 7-KC was also significantly diminished, but like 70 µM 7-KC treated cells. In contrast, the inhibitory effects of 17.5 µM 7-KC treated cells was considerably less, as expected and reported before (Fig. 2B ^26^). Reconstituting plasma membrane of 70 µM 7-KC treated lymph node cells with 35 µM cholesterol did not alter proliferation (p value > 0.5) (Fig. 2B). In contrast, 35 µM cholesterol significantly reconstituted the membrane order of plasma membranes of 35 µM and 17.5 µM 7-KC treated cells proliferation (3 and 1.2-fold increase compared to the cell cultures with cells not reconstituted with cholesterol (p value < 0.001). Reconstitution of membrane order with 17.5 µM cholesterol, the lowest concentration used, showed minimal revival of proliferation in cells pre-treated with 7-KC (70 µM, 35 µM, 17.5 µM). These data show that 35 µM Cholesterol significantly reconstituted proliferation in cells exposed to 35 µM or 17.5 µM 7-KC (Fig. 2B).

Taken together our data demonstrates that cholesterol significantly reconstituted the membrane disorder induced by 7-KC in dose-dependent manner. Importantly, the reduced c-Ova_323–339_ specific proliferative response in 7-KC treated DO11 CD4^+^ T cells was significantly reconstituted after restoration of their membrane order with cholesterol.

## Discussion

Antigen receptor expressed by T lymphocyte, initiate signaling after engaging their membrane-bound ligand(s). Early spatiotemporal signaling events occur in the lipid environment of the plasma membrane. Experimentation with model membranes has demonstrated the self-organizing behavior of phospholipids into liquid ordered (L_o_) and disordered (L_d_) phases where saturated lipids and cholesterol generate order. In contrast, the unsaturated phospholipids promote disorder (L_d_ phase) in these model membranes^5–11^. Existence of the ordered and disordered phases within the plasma membrane of eukaryotic cells is controversial and their contribution to the physiology of cellular responses remains unclear. Moreover, compositional heterogeneity of ordered domains^38,39^, and association of cholesterol with protein present in micro-clusters challenge the cell signaling role of cholesterol-rich ordered domains^40,41^. Much of the evidence for the role of ordered membrane domains in cell signaling has emerged using compounds that generate disorder when inserted into the membrane^26,28,42–45^. Biochemical or genetic approaches to reduce cellular cholesterol has generated empirical evidence in support of the role of membrane order in cell signaling^46–48^. However, a direct evidence for recovery of cell signaling in cells with membrane disorder after reconstituting its membrane order is lacking. Our data demonstrates that cholesterol delivered into the previously disordered membrane can recover its cell signaling through the antigen receptor. These experiments demonstrate a critical role of the cholesterol-dependent membrane order (L_o_) in antigen-specific clonal expansion of CD4^+^ T cells.

Membrane order is contributed by a variety of compositionally heterogeneous membrane domains that are enriched or devoid of cholesterol. While the extensively studied lipid rafts (LRs) enriched in saturated lipids, cholesterol and lipid-anchored proteins are the reported ordered membrane domains^49–52^, the sphingolipid-rich ordered domains without cholesterol are present on plasma membrane contribute to membrane order as well^53^. These ordered domains are heterogeneous in size (10–100 nm) and protein composition^20,50–53^. Creating disorder in membrane by either taking away cholesterol or inserting oxidative forms of cholesterol into the membrane inhibit CD4^+^ T cell responses, and therefore considered as experimental evidence for the role of LRs/LR-based lipid ordered domains, and other ordered membrane domains in T cell signaling and activation^26,28,42–45^. Restoring membrane order domains by inserting cholesterol in disordered membrane create cholesterol-rich ordered domains (Fig. 1), these likely are the cholesterol-dependent LRs and not the sphingolipid-enriched ordered domains devoid of cholesterol. Whether LRs are a subset or encompass all cholesterol-rich ordered membrane domains in T cells is not clear. A complete relationship between the compositionally heterogeneous membrane domains requires further investigation.

Sensing of foreign antigen by the TCRαβ (antigen receptor) and signaling cascade emanating from the plasma membrane initiated by its associated CD3 polypeptides take place in the lipid microenvironment of the plasma membrane. Influences exerted by T cell membrane fluidity with intrinsic order (L_o_) and disorder (L_d_) on early signaling events remain unclear. Role of cholesterol and saturated lipid-rich lipid rafts in compartmentalizing signaling molecules and in contributing to cell signaling has long been proposed^43–45,50^. Lipid rafts and nanodomains are known to contribute to spatiotemporal regulation of cell signaling. Lipid rafts coalesce during sensing of the antigen and are enriched within the immunological synapse^22,28,45^. Interaction between the CD4^+^ T cells with the antigen presenting cells, in antigen-independent manner, promote coalescing of lipid raft domains^24^. T cells with altered levels of cellular cholesterol targeting gene(s) critical for its biosynthesis or regulating cellular cholesterol levels (influx or efflux) alter responsiveness^54^. The effects of altered cholesterol levels, both in the membrane and the cellular organelles is known to contribute to the observed cellular behavior^54^. Direct contribution of membrane lipid order to cellular responsiveness is not known. Limited reach of Di-4ANEPPDHQ fluorescent dye to the outer leaflet of the plasma membrane and its biophysical property to anchor between the polar head groups of lipid molecules with no significant change in its orientation^55^ allowed us to assess membrane fluidity. A published account shows the absence of massive endocytosis (MEND) in cells after incorporating Di-4ANEPPDHQ in the membrane^56^, in contrast, Hela cells show some evidence of dye internalization^34^. To directly assess this issue in our experiments with primary lymphoid cells, we have examined cells under confocal microscope after staining with Di-4-ANEPPDHQ at ambient temperature and compared it with cells stained at 4 °C. These experiments were carried out using using previously described staining and confocal microscopy imaging protocols^34^. While we cannot rule out some internalization of the dye, the bulk staining was observed on the plasma membrane (Fig. 2S). These properties of the dye to interrogate membrane fluidity in quiescent primary lymphoid cells has allowed us to directly assess the antigen-specific T cell signaling role of cholesterol-dependent membrane order within the plasma membrane. Property of this reporter dye to report biochemical reconstitution of the membrane order in combination with the recovery of antigen-specific T cell responsiveness demonstrate the importance of cholesterol in organizing/contributing to membrane L_o_ and cellular responses.

The mechanism how disorder in the membrane disrupts antigen sensing and/or receptor signaling remains unclear. Lipid raft aggregation that potentially brings together the Src kinases and their substrates and its role in downstream signaling events triggered by engaging TCR^51^ is one such possibility. Recent studies show raft coalescence on cellular interactions between CD4^+^ T cells and antigen presenting cell, in an antigen-independent manner^24^ it is posited that this cellular interaction potentially prepares the membrane for upcoming signaling events and T cell activation^26^. Immunological synapse, a site of early T cell activation events located at the contact site of interacting T cell and antigen presenting cell is condensed and appear to be part of ordered domain^28^. Early activation events, in primary CD4^+^ T cells, gauged by activation of p56^lck^ and LAT proteins, are unaffected by disruption of membrane order^28,57^. However, published accounts show possible effects of lipid raft integrity on calcium flux and AKT activation responses^28^. These observations suggest that, in primary T cells, the signaling through the antigen receptor occurs outside the membrane rafts and its role in cell signaling only emerges either at the later parts of the membrane proximal signaling events or downstream of it. Consistent with this idea are the published reports^17^. Additionally, super resolution microscopy has demonstrated monomeric random distribution of antigen receptors on the plasma membrane of naïve T cells^16^. These monomeric antigen receptors trigger signaling by engaging the peptide-MHC complexes outside lipid rafts^17,58^. While these data suggest some spatiotemporal regulation of signaling other reports show specific binding of cholesterol molecules to the β chain of the antigen receptor and role of this interaction in T cell activation and responses^38,39^. Future investigations are required to examine relative contribution of the role of cholesterol associated with the antigen receptor and cholesterol that orchestrates membrane order.

## Conclusions

Spectrally resolved fluorescence of membrane order and disorder by flow cytometer was developed and performed. In here, we demonstrate that by adding back cholesterol to the disordered plasma membrane, the membrane order and disrupted CD4^+^ T cell response is restored. These findings demonstrate that cholesterol-dependent membrane order is critical for responses generated by CD4^+^ T cells and point to the importance of membrane order/fluidity and lipid microenvironment in signaling through T cell membrane antigen receptors.

## Supplementary Information

Supplementary Information.

## Data Availability

All data generated or analyzed during this study are included in this published article. Relevant raw data are available from the corresponding author on reasonable request.

## Abbreviations

L_o_: Membrane ordered phase
L_d_: Membrane disordered phase
7-KC: 7-Ketocholesterol
MβCD: Methyl beta cyclodextrin
CD4: Cluster differentiation antigen 4
TCRαβ: T cell receptor-alpha beta
µM: Micromolar
mM: Millimolar
GTP: Guanine triphosphate
NFκB: Nuclear factor of κ B cells
NFAT: Nuclear factor of activated T cells
AP-I: Activation protein-1
GUV: Giant unilamellar vesicles
GPMV: Giant plasma membrane vesicle
MHC: Major histocompatibility antigens
Di-4-ANEPPDHQ (Di-4-ANE): Polarity dye
IACUC: Institution animal care and use committee
RPMI 1640: Roswell Park Memorial Institute 1640 Medium
HEPES: (4-(2-Hydroxyethyl)-1-piperazineethanesulfonic acid)
FL 1–4: Fluorescence channels 1–4
rGP: Ratiometric generalized polarization
MFI: Mean fluorescence Intensity
MTT: 3-(4,5-Dimethylthiazol-2-yl)-2,5-diphenyl tetrazolium bromide
c-Ova_323–339_: Chicken ovalbumin peptide sequence 323–339
ANOVA: Analyses of variance
LAT: Linker of activated T cells

## References

[1] Malissen B, Bongrand P (2015). Early T cell activation integrating biochemical structural and biophysical cues. Annu. Rev. Immunol..

[2] Courtney AH, Lo WL, Weiss A (2018). TCR signaling: Mechanisms of initiation and propagation. Trends Biochem. Sci..

[3] Gaud G, Lesourne R, Love PE (2018). Regulatory mechanisms in T cell receptor signaling. Nat Rev Immunol..

[4] Heinzel S, Marchingo JM, Horton MB, Hodgkin PD (2018). The regulation of lymphocyte activation and proliferation. Curr. Opin. Immunol..

[5] Korlach J, Schwille P, Webb WW, Feigenson GW (1999). Characterization of lipid bilayer phases by confocal microscopy and fluorescence correlation spectroscopy. Proc. Natl. Acad. Sci. USA..

[6] Veatch SL, Keller SL (2003). Separation of liquid phases in giant vesicles of ternary mixtures of phospholipids and cholesterol. Biophys. J..

[7] Sankaram MB, Thompson TE (1991). Cholesterol-induced fluid-phase immiscibility in membranes. Proc. Natl. Acad. Sci. USA.

[8] Dietrich C (2001). Lipid rafts reconstituted in model membranes. Biophys. J..

[9] de Meyer F, Smit B (2009). Effect of cholesterol on the structure of a phospholipid bilayer. Proc. Natl. Acad. Sci. USA.

[10] Silvius JR (2003). Role of cholesterol in lipid raft formation: lessons from lipid model systems. Biochim. Biophys. Acta..

[11] Levental KR, Levental I (2015). Giant plasma membrane vesicles: Models for understanding membrane organization. Curr. Top. Membr..

[12] Munro S (2003). “Lipid rafts: Elusive or illusive?. Cell.

[13] Shaw AS (2006). Lipid rafts: Now you see them, now you don't. Nat. Immunol..

[14] Edidin M (2003). The state of lipid rafts: From model membranes to cells. Annu. Rev. Biophys. Biomol. Struct..

[15] Kenworthy AK (2008). Have we become overly reliant on lipid rafts?. EMBO Rep..

[16] Brameshuber M (2018). Monomeric TCRs Drive T Cell Antigen Recognition. Nat. Immunol..

[17] Douglass AD, Vale RD (2005). Single-molecule microscopy reveals plasma membrane microdomains created by protein-protein networks that exclude or trap signaling molecules in T cells. Cell.

[18] Harder T (2004). Lipid raft domains and protein networks in T-cell receptor signal transduction. Curr Opin Immunol..

[19] Kusumi A, Koyama-Honda I, Suzuki K (2004). Molecular dynamics and interactions for creation of stimulation-induced stabilized rafts from small unstable steady-state rafts. Traffic.

[20] Mayor S, Rao M (2004). Rafts: Scale-dependent, active lipid organization at the cell surface. Traffic.

[21] Hancock JF (2006). Lipid rafts: Contentious only from simplistic standpoints. Nat. Rev. Mol. Cell. Biol..

[22] Viola A, Gupta N (2007). Tether and trap: Regulation of membrane-raft dynamics by actin-binding proteins. Nat. Rev. Immunol..

[23] Day CA, Kenworthy AK (2009). Tracking microdomain dynamics in cell membranes. Biochim. Biophys. Acta..

[24] Kennedy C, Nelson MD, Bamezai AK (2011). Analysis of detergent-free lipid rafts isolated from CD4^+^ T cell line: interaction with antigen presenting cells promotes coalescing of lipid rafts. Cell Commun. Signal..

[25] Murphy KM, Heimberger AB, Loh DY (1990). Induction by antigen of intrathymic apoptosis of CD4^+^CD8^+^TCR^lo^ thymocytes in vivo. Science.

[26] Schieffer D, Naware S, Bakun W, Bamezai AK (2014). Lipid raft-based membrane order is important for antigen-specific clonal expansion of CD4^+^ T lymphocytes. BMC Immunol..

[27] Zidovetzki R, Levitan I (2007). Use of cyclodextrins to manipulate plasma membrane cholesterol content: Evidence, misconceptions and control strategies. Biochim. Biophys. Acta Biomembr..

[28] Rentero C (2008). Functional implications of plasma membrane condensation for T cell activation. PLoS ONE.

[29] Jin L (2006). Characterization and application of a new optical probe for membrane lipid domains. Biophys. J..

[30] Dinic J, Biverståhl H, Mäler L, Parmryd I (2011). Laurdan and di-4-ANEPPDHQ do not respond to membrane-inserted peptides and are good probes for lipid packing. Biochim. Biophys. Acta..

[31] Owen DM, Gaus K (2010). Optimized time-gated generalized polarization imaging of Laurdan and di-4-ANEPPDHQ for membrane order image contrast enhancement. Microsc. Res. Tech..

[32] Gaus K (2003). Visualizing lipid structure and raft domains in living cells with two-photon microscopy. Proc. Natl. Acad. Sci..

[33] Gaus K, Zech T, Harder T (2006). Visualizing membrane microdomains by Laurdan 2-photon microscopy. Mol. Membr. Biol..

[34] Owen DM, Rentero C, Magenau A, Abu-Siniyeh A, Gaus K (2011). Quantitative imaging of membrane lipid order in cells and organisms. Nat Protoc..

[35] Berridge MV, Tan AS (1993). Characterization of the cellular reduction of 3-(4,5-dimethylthiazol-2-yl)-2,5-diphenyltetrazolium bromide (MTT): Subcellular localization, substrate dependence, and involvement of mitochondrial electron transport in MTT reduction. Arch. Biochem. Biophys..

[36] Pearce E (2010). Metabolism in T cell activation and differentiation. Curr. Top. Immunol..

[37] Chapman NM, Boothby MR, Chi H (2020). Metabolic coordination of T cell quiescence and activation. Nat. Rev. Immunol..

[38] Pike LJ (2003). Lipid rafts: bringing order to chaos. J. Lipid. Res..

[39] George S, Nelson MD, Dollahon N, Bamezai A (2006). A novel approach to examining compositional heterogeneity of detergent-resistant lipid rafts. Immunol. Cell Biol..

[40] Swamy M (2016). A cholesterol-based allostery model of T cell receptor phosphorylation. Immunity.

[41] Wang J, Megha E, London E (2004). Relationship between sterol/steroid structure and participation in ordered lipid domains (lipid rafts): Implications for lipid raft structure and function. Biochemistry.

[42] Kabouridis PS, Janzen J, Magee AL, Ley SC (2000). Cholesterol depletion disrupts lipid rafts and modulates the activity of multiple signaling pathways in T lymphocytes. Eur. J. Immunol..

[43] Xavier R, Brennan T, Li Q, McCormack C, Seed B (1998). Membrane compartmentation is required for efficient T cell activation. Immunity.

[44] Montixi C (1998). Engagement of T cell receptor triggers its recruitment to low-density detergent-insoluble membrane domains. EMBO J..

[45] Janes PW, Ley SC, Magee AI (1999). Aggregation of lipid rafts accompanies signaling via the T cell antigen receptor. J. Cell Biol..

[46] Gimpl G, Burger K, Fahrenholz F (1997). Cholesterol as modulator of receptor function. Biochemistry.

[47] Yang W (2016). Potentiating the anti-tumor response of CD8^+^ T cells by modulating cholesterol metabolism. Nature.

[48] Miguel L (2011). Primary human CD4^+^ T cells have diverse levels of membrane lipid order that correlate with their function. J. Immunol..

[49] Simons K, Ikonen E (1997). Functional rafts in cell membranes. Nature.

[50] Brown DA, London E (1998). Functions of lipid rafts in biological membranes. Annu. Rev. Cell Dev. Biol..

[51] Viola A, Schroeder S, Sakakibara Y, Lanzavecchia A (1999). T Lymphocyte costimulation mediated by reorganization of membrane microdomains. Science.

[52] Simons K, Gerl MJ (2010). Revitalizing membrane rafts: New tools and insights. Nat. Rev. Mol. Cell. Biol..

[53] Frisz JF (2013). Sphingolipid domains in the plasma membranes of fibroblasts are not enriched with cholesterol. J. Biol. Chem..

[54] Bensinger SJ (2008). LXR signaling couples sterol metabolism to proliferation in the acquired immune response. Cell.

[55] Suhaj A (2020). Laurdan and di-4-ANEPPDHQ influence the properties of lipid membranes: A classical molecular dynamics and fluorescence study 2020. J. Phys. Chem. B.

[56] Hilgemann DW, Fine M (2011). Mechanistic analysis of massive endocytosis in relation to functionally defined surface membrane domains. J. Gen. Physiol..

[57] Bamezai AK, Bakun W (2016). Role of lipid raft-based membrane order in signaling through the antigen receptor in CD4^+^ T cells: Investigating the mechanism. J. Immunol..

[58] Rossboth B (2018). TCRs are randomly distributed on the plasma membrane of resting antigen-experienced T cells. Nat. Immunol..

