# Supplementary material for: Cholesterol-dependent plasma membrane order (Lo) is critical for antigen-specific clonal expansion of CD4+ T cells
Source: Sci Rep. 2021 Jul 7;11:13970. doi: 10.1038/s41598-021-93403-5 (PMC8263698; doi:10.1038/s41598-021-93403-5)
Supplement: Supplementary file 1 — Supplementary Information. [file 41598_2021_93403_MOESM1_ESM.pdf]

Cholesterol-dependent plasma membrane order (L_o) is critical for antigen-specific clonal expansion of CD4$^+$ T cells

Soumini Sengupta[1,2], Ritesh Karsalia[1,3], Amanda Morrissey[1,4] and Anil K Bamezai[1*]

[1]Department of Biology, Villanova University,

800E Lancaster Avenue, Villanova, PA 19085 USA.

[*]Corresponding Author E-mail: anil.bamezai@villanova.edu

[2]E-mail: ssengupt@villanova.edu

[3]E.mail: rkarsali@villanova.edu

[4]E-mail: amorri26@villanova.edu

Running Title: Role of membrane ordered phase (L_o) in CD4$^+$ T cell response.

Supplementary figure 1

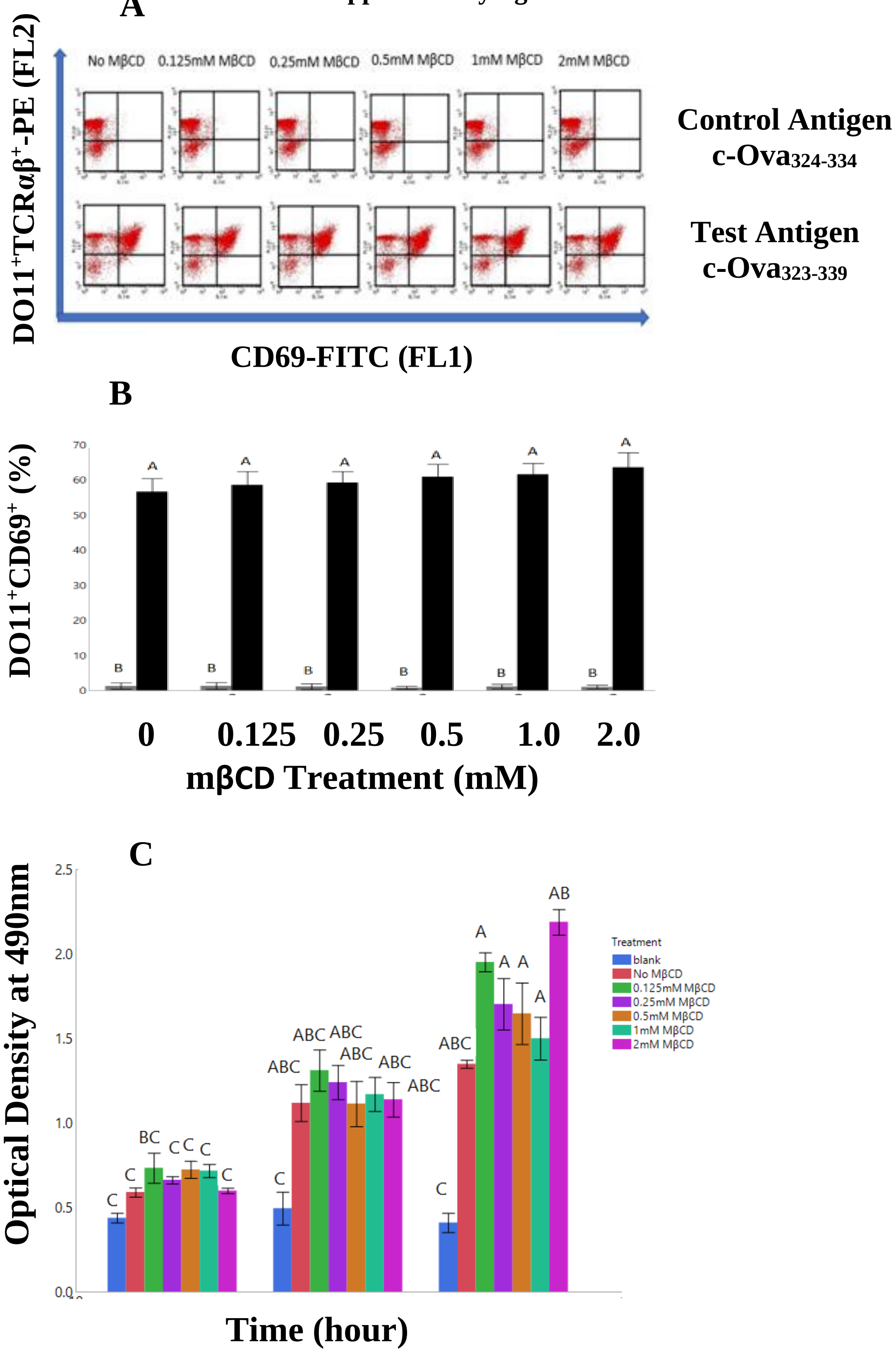

Supplementary Figure 1: Effect of MβCD on antigen-specific response by $CD4^+$ T cells. DO11 TCR transgenic lymph node cells were treated with MβCD, at concentrations ranging from 0.125 mM to 2 mM for 10 minutes at RT, followed by stimulation with c-$Ova_{323-339}$ (test) and c-$Ova_{324-334}$ (control peptide) for 24 hours at 37°C. To enumerate activated T cells, cells were then stained with anti-CD69-FITC and anti DO11TCRαβ-PE for 45 minutes on ice and live cells were examined for the expression of CD69 and DO11TCRαβ by FACS Calibur (A). Average percent $CD69^{+}DO11^{+}$ TCRαβ cells in the live population in control and test cultures is shown (n=5) (B). To assess effects of MβCD on antigen-specific proliferation of DO11 T cells, lymph node cells were treated with different concentrations of MβCD as above and cultured with Chicken Ovalbumin $_{323-339}$ for 24, 48 and 72 hours at 37°C. 20µl of MTT reagent was added during last 4 hours incubation and microtiter plates were read in a plate reader at 490 nm (C). Average optical density with error bars from 5 independent trials is shown (C). Statistical significance between the groups was computed by two-way ANOVA and post hoc Tukey analysis using JMP program. Groups with dissimilar connecting letters are significantly different from each other. MβCD concentrations used did not alter GP value compared with untreated control (Figure 1B and data not shown)

Figure S2

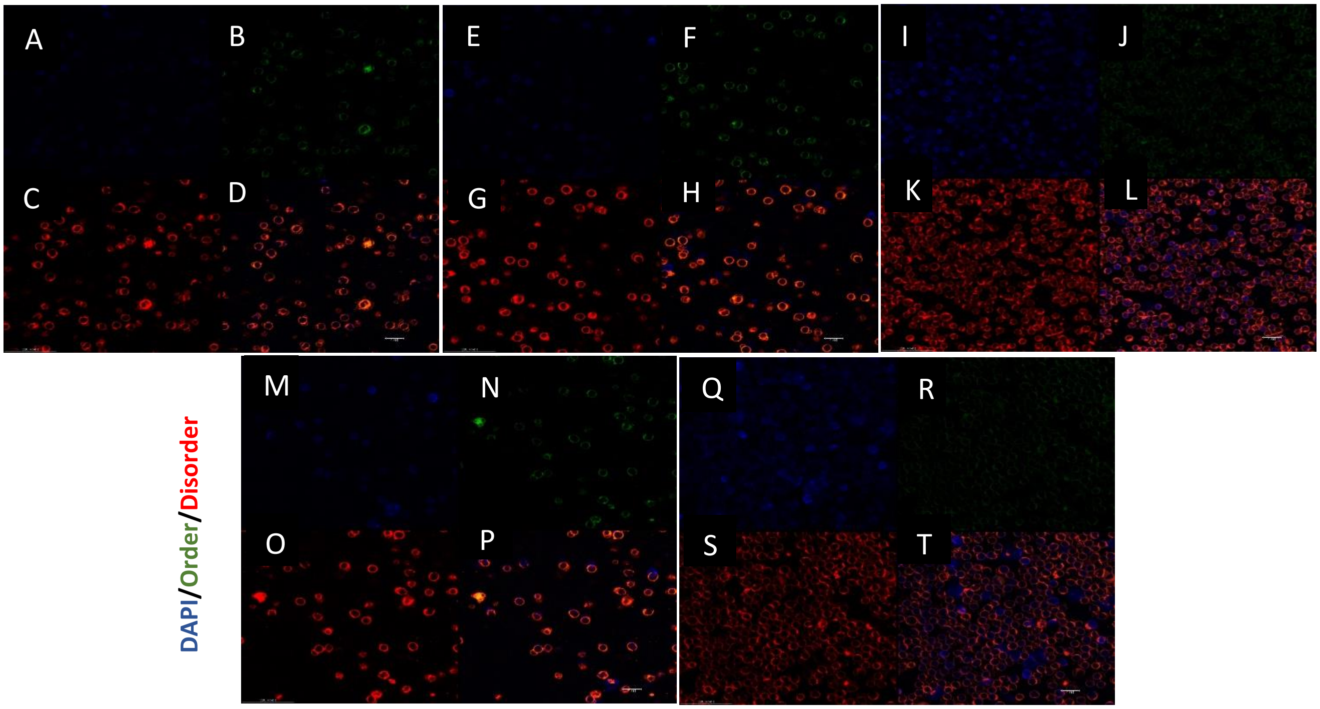

Figure 2S. Live cell imaging of Di-4-ANEPPDHQ stained cells by confocal microscope: DO11 TCRαβ Transgenic mouse lymph node T cells adhered to poly-L-lysine coated plates were not treated (A-D) or treated with 35μM 7KC (E-H) for 10 minutes and then stained with Di-4ANEPPDHQ either at RT (A-H) or control 4°C (M-T). As additional control, cells were first fixed with 4% paraformaldehyde phosphate buffer solution for 5 minutes at room temperature before staining with Di-4ANEPPDHQ (I-L). Slides were mounted with mounting media containing DAPI (Vector Labs) and covered with a glass coverslip with edges sealed using clear nail polish. Prepared slides were visualized by confocal microscope immediately after staining and stored on ice prior to and during confocal imaging to prevent dye internalization. Cells were imaged using a Leica TCS SP8 inverted confocal fluorescence microscope equipped with standard and HyD PMT's at 630x total magnification and set to a sequential scan. Samples were illuminated with 408 nm (DAPI) and 488 nm (Di-4-ANEPPDHQ) lasers. DAPI (Blue)

Figure S2

was detected with a standard PMT with a wavelength range of 410-460 nm. Di-4-ANEPPDHQ was collected with two HyD PMTs, the first detecting wavelengths in the range of 500-540 nm (Ordered Phase). The second HyD PMT collected wavelengths in the 640 – 750 nm range (Disordered Phase). The image size was 512 x 512 pixels and pinhole adjusted to 1 Airy Unit. Scan speed was 400 Hz and line average set at 4. Gains for HyD2 (Ordered-green) and HyD3 (Disordered-red) were set to a moderate level. Zoom was set to 1. The Ordered phase was pseudo-colored green (upper right panels in each – B, F, J, N, R) while the Disordered phase was pseudo-colored red (lower left panel in each – C, G, K, O, S) and DAPI-stained nucleus as blue (upper left panel – A, E, I, M, Q). Overlayed staining of the ordered, disordered and nucleus is shown (lower right panels – D, H, L, P, T). The equipment setup was optimized from the protocol described by Owen et al., 2012. Magnification 63X; Scale bar, shown as 4.1µm (right) and 200 pixels (left) is shown. A representative immunofluorescence images of two independent experiments are shown.
